# Evidence for B Cell Exhaustion in Chronic Graft-versus-Host Disease

**DOI:** 10.3389/fimmu.2017.01937

**Published:** 2018-01-12

**Authors:** Ahmad Khoder, Abdullah Alsuliman, Rafet Basar, Catherine Sobieski, Kayo Kondo, Amin Majid Alousi, Richard Szydlo, Muharrem Muftuoglu, Hila Shaim, Jane F. Apperley, Elif Gokdemir, Nichola Cooper, Rohtesh S. Mehta, David Marin, Richard Champlin, Elizabeth Shpall, Katayoun Rezvani

**Affiliations:** ^1^Department of Haematology, Imperial College London, London, United Kingdom; ^2^Stem Cell Transplantation and Cellular Therapy, University of Texas MD Anderson Cancer Center, Houston, TX, United States

**Keywords:** chronic graft-versus-host disease, CD19+CD21−CD27−CD10− cells, exhausted B cells, stem cell transplantation, CD21– B cells correlate with cGVHD severity

## Abstract

Chronic graft-versus-host disease (cGvHD) remains a major complication of allogeneic hematopoietic stem cell transplantation (HSCT). A number of studies support a role for B cells in the pathogenesis of cGvHD. In this study, we report the presence of an expanded population of CD19+CD21− B cells with features of exhaustion in the peripheral blood of patients with cGvHD. CD21− B cells were significantly increased in patients with active cGvHD compared to patients without cGvHD and healthy controls (median 12.2 versus 2.12 versus 3%, respectively; *p* < 0.01). Compared with naïve (CD27−CD21+) and classical memory (CD27+CD21+) B cells, CD19+CD21− B cells in cGvHD were CD10 negative, CD27 negative and CD20hi, and exhibited features of exhaustion, including increased expression of multiple inhibitory receptors such as FCRL4, CD22, CD85J, and altered expression of chemokine and adhesion molecules such as CD11c, CXCR3, CCR7, and CD62L. Moreover, CD21− B cells in cGvHD patients were functionally exhausted and displayed poor proliferative response and calcium mobilization in response to B-cell receptor triggering and CD40 ligation. Finally, the frequencies of circulating CD21− B cells correlated with cGvHD severity in patients after HSCT. Our study further characterizes B cells in chronic cGVHD and supports the use of CD21−CD27−CD10− B cell frequencies as a biomarker of disease severity.

## Introduction

Chronic graft-versus-host disease (cGvHD) remains a major limiting factor to the success allogeneic hematopoietic stem cell transplantation (HSCT). Chronic GvHD occurs in 30–65% of HSCT recipients with a 5-year mortality rate of up to 30–50% (1). At present, the immunopathophysiology of cGVHD is not fully understood, thereby hindering the development of effective treatments. Traditionally, cGvHD has been approached as a T cell-mediated process and many of the current therapies reflect this understanding (2). However, recent clinical and preclinical studies also suggest a role for B cells in the pathogenesis of cGvHD (3–5).

The occurrence of cGvHD in the settings of major or minor histocompatibility antigen disparity and the identification of multiple autoantibodies imply either a direct breakdown in the B cell self-tolerance process or the existence of aberrant B cell responses to T cell help (6, 7). Patients with cGvHD have a number of abnormalities in their B cell compartment, including lower frequencies of marrow B cell precursors (8–11), fewer regulatory B cells (12–14), a skewed immunoglobulin repertoire (9, 15), as well as significantly fewer peripheral blood (PB) CD27+ B cells compared to patients without cGvHD (16–19). In addition, memory B cells from patients with cGvHD are activated and in a higher metabolic state, with increased signaling through the AKT and extracellular signal-regulated kinase pathway (20), which, in association with elevated plasma B cell-activating factor (BAFF) to B-cell ratio, may promote the aberrant survival of autoreactive B cells (21, 22). In addition, higher frequencies of CD19+CD21− B cells have been observed in the PB of patients with cGvHD. The authors identified these cells as transitional B cells (16, 23), and associated their presence with bronchiolitis obliterans syndrome (BOS) (24). Moreover, CD19+CD21low B cells frequencies measured on day 100 following allogeneic HCT were found to predict for the development of cGVHD (25).

Here, we report on the presence of an expanded population of CD19+CD21− B cells in patients with active cGvHD. We further characterize this population phenotypically and functionally and show similarities with the exhausted B cells described in chronic HIV viremia (26). These features include increased expression of multiple inhibitory receptors, altered expression of chemokine and adhesion molecules, as well as poor proliferative response to a variety of stimuli. These data may in part explain the inadequacy of the antibody response against pathogens and the state of immunodeficiency observed in cGVHD patients.

## Materials and Methods

### Inclusion Criteria

All patient samples were collected after written informed consent, on research protocols approved by the Institutional Review Boards at MD Anderson Cancer Center, Houston, TX, USA and the Hammersmith Hospital, London England, according to local policy guidelines and in accordance with the declaration of Helsinki.

Peripheral blood samples were obtained from 55 patients who had undergone allogeneic HSCT between 1998 and 2011, had no evidence of primary disease relapse and were 6 months or more post-HSCT. The cohort were selected randomly and included patients who underwent reduced intensity or myeloablative conditioning and received bone marrow or mobilized peripheral blood stem cell grafts from an HLA-matched sibling or unrelated donor. Chronic GvHD status at the time of sample collection was classified according to documented clinical and laboratory data using National Institutes of Health (NIH) cGvHD consensus criteria and the modified Seattle criteria for limited versus extensive disease (27, 28). Patients receiving high-dose steroids (>0.75 mg/kg prednisone) at the time of sampling or those who had received rituximab for any indication were excluded.

### Processing of Peripheral Blood Mononuclear Cells (PBMC)

Peripheral blood mononuclear cells were separated from whole blood by density gradient centrifugation (Ficoll-Hypaque, Durham, NC, USA), cryopreserved in 20% DMSO in fetal calf serum, and stored in liquid nitrogen. Prior to each experiment, cells were thawed and rested overnight in RPMI 1640 media (Gibco, UK) supplemented with 10% fetal calf serum.

### Classification of B Cell Subsets

Multiparameter flow cytometry was employed to assess B cell phenotype, using a 9-color panel of conjugated mouse antihuman monoclonal antibodies (mAb) specific for the following markers: APC-H7 CD19, FITC CD24, PE CD27, PerCP-Cy5.5 IgM, and v450 IgD (all from BD Pharmingen™ and BD Horizon™, UK), APC CD21, PE-Cy7 CD38 (eBioscience, UK), and Qdot 605 CD10 from (Invitrogen™, USA). PBMC were stained first with 1 µl of the viability marker LIVE/DEAD Fixable Aqua Dead cell stain (L_D), invitrogen™, for 30 min in the dark at room temperature. Cells were then washed and incubated with the cocktail of mAb at titrated concentrations in PBS for a further 30 min at room temperature. Cells were acquired on BD LSRFortessa cell analyzer (BD, Bioscience, USA). Data were analyzed using FlowJo software (Treestar, San Carlos, CA, USA). Fluorescence minus one and unstained controls were used for each fluorochrome to set gates and define positive expression.

Using CD19, CD27, and IgM co-expression, B cell subsets were defined as naïve (CD19+IgM+CD27−) and classical memory B cells (CD19+CD27+). The latter was further subdivided into IgM memory (CD19+CD27+IgM+) and class-switched (CD19+CD27+IgM−) memory B cells. A second phenotypic definition based on expression of CD19, CD24, and CD38 was also employed to define transitional (CD19+CD24hiCD38hi), naïve (CD19+CD24+CD38+), memory (CD19+CD24hiCD38−) and plasmablasts (CD19+CD24−CD38hi).

### Expression of Inhibitory and Chemotaxis Receptors on B Cell Subpopulations

We assessed the expression of inhibitory and trafficking receptors on B cell subpopulations using multi-color flow cytometry using the gating strategy presented in Figure S1 in Supplementary Material. Conjugated mouse antihuman monoclonal mAb specific for the following markers were employed: FITC CD22, PE CD27, PE CD20, PE CD85J, PE-Cy7 CCR6, APC-H7 CD19, and PerCP-Cy5.5 CXCR3 all from (BD Pharmingen™, UK); PE FCRL4, PE-y7 CD62L, and PerCP-Cy5.5 CD11c from (BioLegend, San Diego, CA, USA); APC CD21 from (eBioscience, UK) and Qdot 605 CD10 from (Invitrogen™, USA). Cells were acquired on BD LSRFortessa cell analyzer (BD, Bioscience, USA) and data were analyzed using FlowJo software (Treestar, San Carlos, CA, USA). Median fluorescence intensity (MFI) was used to compare the expression of inhibitory and chemokine receptors in the different study groups.

### Proliferation Assay Using Carboxyfluorescein Succinimidyl Ester (CFSE)

The proliferative capacity of CD19+ B cells was assessed by measuring CFSE dilution after *in vitro* stimulation. In brief, thawed PBMC were rested overnight and stained with CFSE (eBioscience, UK) according to the manufacturer’s protocol. Cells were plated at 2 × 10^5^ cells per well of a 96-well flat-bottom plate with 1 µg/ml of F(ab’) 2 Goat anti Human IgG + IgM (H + L) (Jackson ImmunoResearch Laboratories, USA) alone, 1 µl per 4 × 10^5^ cells of anti-CD3/28 DynaBeads (Invitrogen, Oslo, Norway) alone or in combination. Cells were harvested, washed, and stained with PerCP CD4 and APC CD19 mAbs (both from BD Pharmingen™, UK). CFSE is usually detected at 530/30 nm. Samples were acquired on FACSCalibur (BD Bioscience, Oxford, UK) and data were analyzed using FlowJo software (Treestar, San Carlos, CA, USA).

### Calcium Mobilization Assay

Calcium mobilization assay was carried out according to published protocol (29). Briefly, a total of 3–5 × 10^6^ cells were suspended in dye loading buffer containing 1 µM Ca2+ and 1 µM Mg2+ ions, supplemented with 1% BSA, 0.2% pluronic F-127 (Sigma-Aldrich), and 5 µM Fluo-4-acetoxymethyl ester (Fluo-4-AM) (Invitrogen) for 25 min at 37°C. Cells were subsequently stained with anti-CD19 APC-H7, anti-CD27 PE, and anti-CD21 APC mAbs and resuspended at a concentration of 10^6^ cells/ml. Intracellular calcium in gated CD19+CD27+CD21+ and CD19+CD27+CD21− B cells was monitored over time by flow cytometry. Resulting emission was measured first for 5 min to establish a baseline, and subsequently, 20 µg/ml of goat F (ab′) 2 Goat anti Human IgG + IgM (Jackson ImmunoResearch Laboratories) was added and emission were obtained. Ratios of B-cell subsets MFI at baseline and at 120 s were calculated using the FlowJo software (Treestar, San Carlos, CA, USA). The ratio of intracellular Ca+ 2 MFI at 120 s to baseline MFI was compared in the CD21− and CD21+ B cell populations using the non-parametric paired *T* test.

### Statistical Analysis

Groups were compared using either the Mann–Whitney or Chi square test. For multiple comparisons, the Kruskall–Wallis test with Dunn’s posttest was used. The association of CD21− B cells with cGvHD was investigated using logistic regression analysis, taking into account all variables from the univariate analysis with *p*-values <0.10. All statistical analyses were performed using GraphPad Prism version 5.04 for Windows (GraphPad Software, La Jolla, CA, USA) and IBM SPSS Statistics for Windows, Version 24.0. Armonk, NY, USA. All tests were considered significant when the two-tailed *p-*values <0.05.

## Results

### Patient Characteristics

Patient characteristics are summarized in Table 1. Thirty two patients had “active cGvHD” (limited or extensive) at the time of PB collection. Twenty three patients had no history of cGvHD after HSCT. Seventeen patients were on low dose prednisolone (≤0.75 mg/kg) either alone or in combination with other immunosuppressants, including mycophenolate mofetil (3/32), sirolimus (4/32), or tacrolimus (12/32). Two patients were receiving extracorporeal photopheresis. None of the “no cGvHD” patients were on immunosuppressive therapy at the time of PB collection. Median time to analysis was 543 days (180–4,225) for the cGvHD and 1,107 days (349–4,000) for the no cGvHD group (*p* = 0.007). We also studied 18 age-matched healthy controls (HC).

**Table 1:** Patient, disease, and transplant characteristics.

	No cGvHD (*N* = 23)	cGvHD (*N* = 32)	*p*-Value
Age in years (range)	55 (24–66)	53 (24–68)	0.65
Time post-SCT, days (range)	543 (349–4,000)	1,107 (180–2,947)	0.007

**Patient sex**			
Male (%)	14 (60)	22 (68)	0.54
Female (%)	9 (40)	10 (32)	

**Donor sex**			
Male (%)	19 (83)	19 (59)	0.066
Female (%)	4 (17)	13 (41)	

**Disease**			
Lymphoma and myeloma (%)	2 (7)	13 (41)	
Acute leukemia (%)	17 (74)	13 (41)	
Myelofibrosis (%)	0 (0)	2 (6)	

**Conditioning regimen**			
Myeloablative conditioning (%)	8 (35)	6 (19)	0.18
Reduced intensity conditioning (%)	15 (65)	26 (81)	

**Stem cell donor**			
Related (%)	7 (30)	24 (75)	0.001
Unrelated (%)	16 (70)	8 (25)	

**Stem cell source**			
Bone marrow (%)	8 (36.5)	4 (13)	0.6
Peripheral blood stem cells (%)	14 (63.5)	29 (87)	
CD34+ cells ×106/kg	4.99 (0.79–12.2)	6.25 (2.8–12.77)	0.25

**Posttransplant immunosuppressive prophylaxis**											
CsA + MTX (%)	6 (26)	7 (22)	0.83
CsA + MTX—T cell antibodies (%)	6 (26)	7 (22)	
Tacrolimus-MTX (%)	11 (48)	18 (46)	

**Acute GVHD**			
Grade 0 (%)	13 (56)	16 (50)	0.4
Grades I–II (%)	8 (35)	11 (34)	
Grades III–IV (%)	2 (9)	5 (16)	

**Donor lymphocyte infusion (DLI)**															
Received DLI (%)	5 (22)	10 (31)	0.43
Received no DLI (%)	18 (78)	22 (69)	

*MTX, methotrexate; CsA, cyclosporine; DLI, donor lymphocyte infusion; cGvHD, chronic graft-versus-host disease*.

### Chronic GvHD Is Associated with Decreased Numbers of Naïve and Transitional and Higher Frequencies of CD27+ Memory B Cells

**Figure 1:**
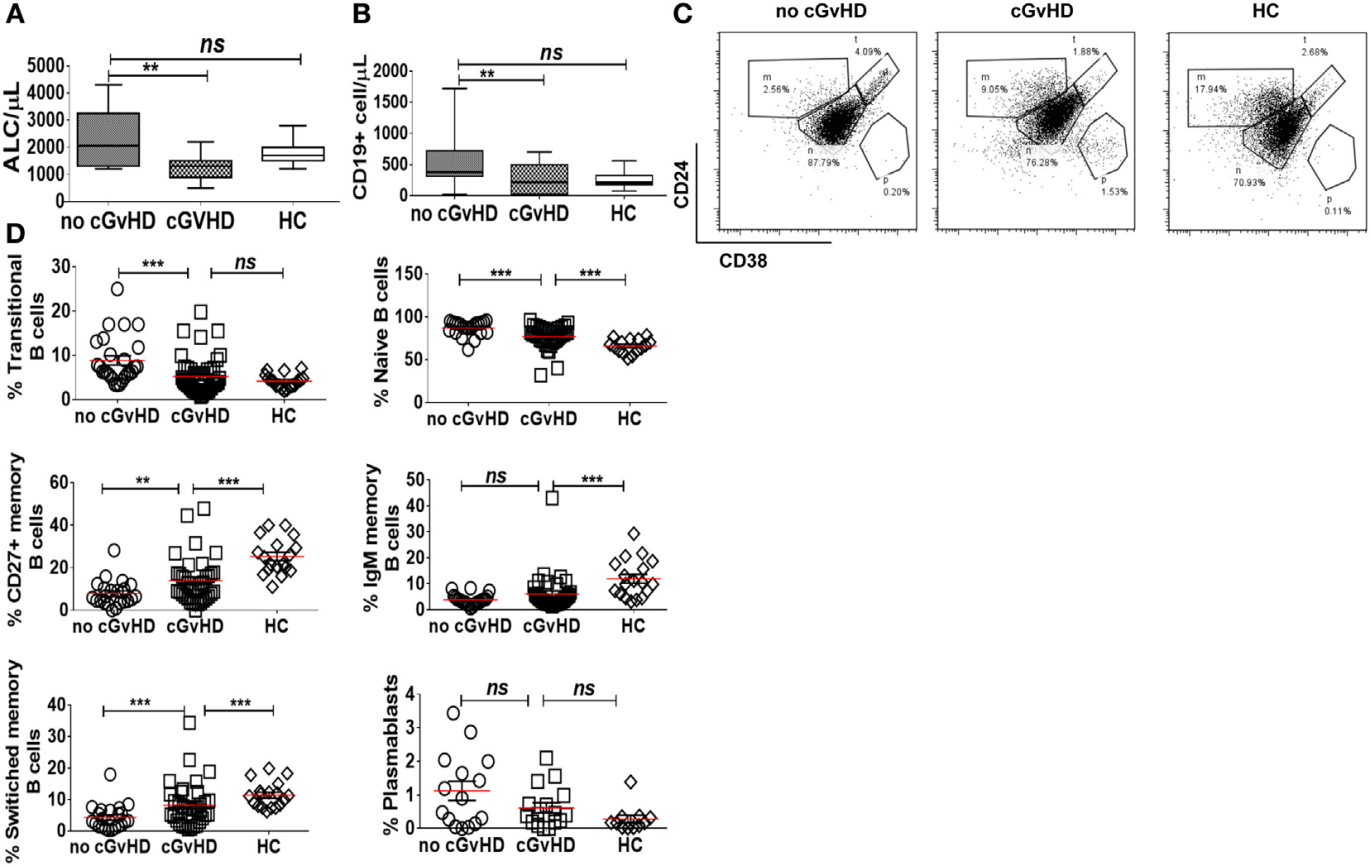
B cell subsets in patients with or without chronic graft-versus-host disease (cGvHD) and healthy controls (HC). Box plots, showing medians, 25th and 75th percentiles and ranges, compare total lymphocyte count **(A)** and total CD19+ B cell count **(B)** in cGvHD patients compared to “no cGvHD” patients and HC. **(C)** Representative FACS plots demonstrating CD19+ B cell phenotype in a “no GvHD,” a cGvHD patient and a healthy control. Gated CD19+ B cells were examined for CD24 and CD38 expression; transitional (CD19+CD24hiCD38hi), naïve (CD19+CD24+CD38+) and memory (CD19+CD24hiCD38−) B cell subsets are presented. **(D)** Scatter plots compare the percentage of individual B cell subsets in patients with “no GvHD” (*n* = 23), with cGvHD (*n* = 32) and HC (*n* = 18).

Consistent with previously published studies (18), patients with cGvHD in our study had significantly lower absolute lymphocyte counts than patients without cGvHD and HC (median 1,300 versus 2,050 versus 1,700 cell/μl; *p* = 0.008 and *p* = 0.01, respectively); Figure 1A. Total CD19+ B cell numbers were also significantly lower in patients with cGvHD compared to patients without cGvHD (median 216 versus 378 cell/μl; *p* = 0.002); Figure 1B. However, patients without cGvHD had higher CD19+ B cells compared with HC (median 378 versus 180 cells/μl; *p* = 0.013), consistent with the previously described B cell “surge” following HSCT (9, 21).

Next, we employed multiparameter flow cytometry to examine B cell subsets in post-HSCT patients and controls. In agreement with previously published data (22), patients with active cGvHD had significantly fewer transitional (CD19+CD24hiCD38hi) and naïve B cells (CD19+CD24+CD38+) compared to patients without cGvHD (median 0.5 versus 22.3 cell/μl; *p* = 0.08 and 158 versus 338 cell/μl; *p* = 0.03, respectively). In the setting of relative naive B lymphopenia, patients with cGvHD had proportionally higher frequencies of CD27+CD19+ B cells (15.6% in active cGvHD versus 6.6% in patients without cGvHD; *p* = 0.01 and 25% in HC; *p* = 0.0001), Figures 1C,D. The absolute CD27+CD19+ B cell count in the PB of patients post-HSCT was significantly lower compared to HC, regardless of cGvHD status (data not shown).

### Chronic GvHD Patients Have Higher Frequencies of Circulating CD19+CD21− B Cells

**Figure 2:**
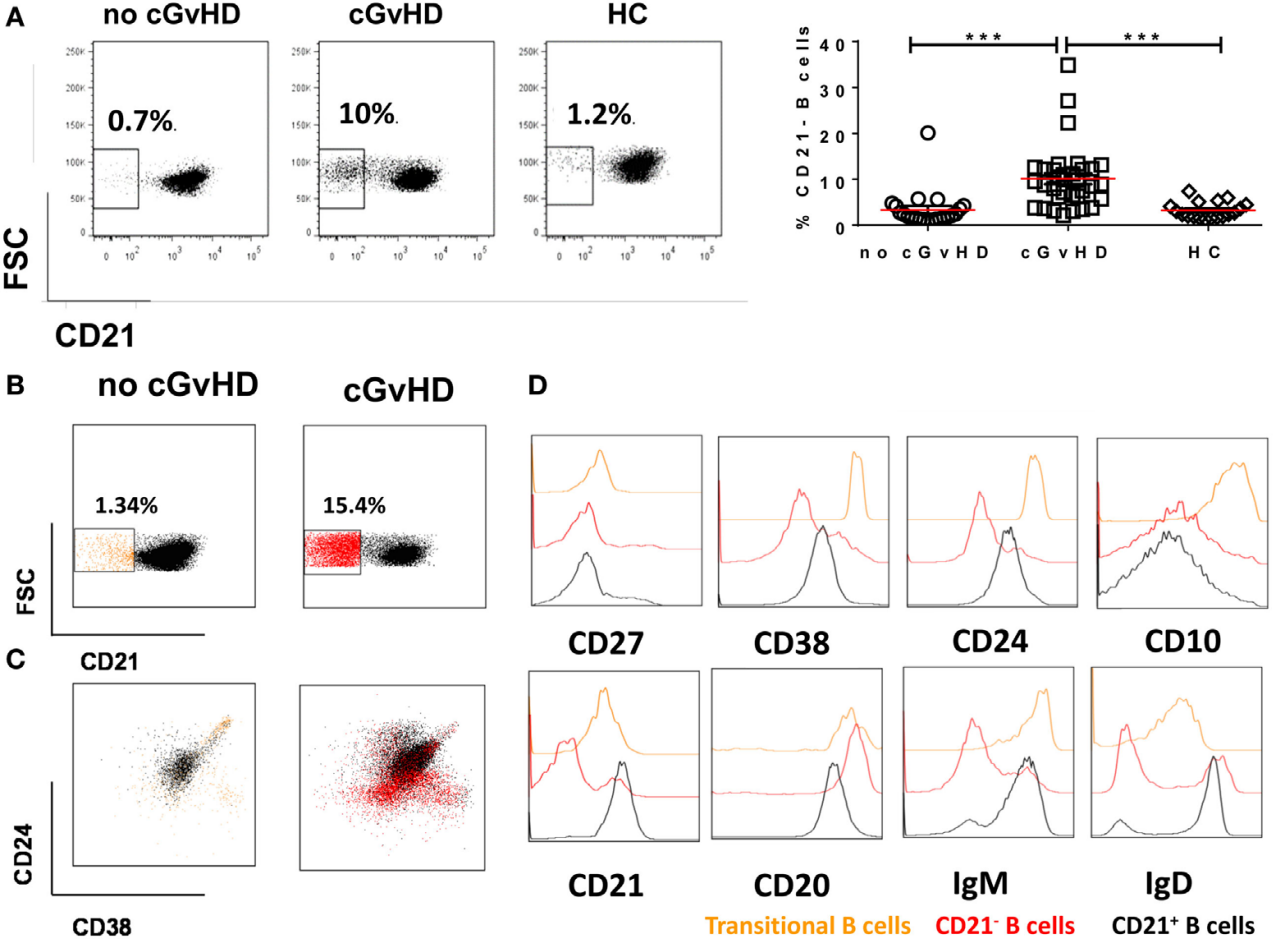
CD19+CD21− B cells in patients with or without chronic graft-versus-host disease (cGvHD) and healthy controls (HC). **(A)** FACS plots showing CD21− B cell frequency in a representative “no GvHD” patient, GvHD patient, and a healthy control. Chronic GvHD patients (*n* = 32) have significantly higher percentage of circulating CD21− B cells compared with “no cGvHD” patients (*n* = 23) and HC (*n* = 18). **(B,C)** Color-coded gating on CD19+ B cells in representative “no GvHD” and active cGvHD patients; **(B)** cGvHD patient has higher frequencies of CD21− B cells (red) compared with the “no cGvHD” representative patient (yellow). **(C)** Gated CD19+ B cells from the same examples are shown; the majority of CD21− B cells in the “no cGvHD” group (yellow) fall within the CD24hiCD38hi transitional B cell and CD24−CD38hi plasmablast regions. Conversely, the majority of CD21− B cells in “cGvHD” patients (Red) fall within the CD24−CD38− exhausted B cell and CD24−CD38hi plasmablast regions. **(D)** Phenotypic characterization of transitional (yellow) and exhausted CD21− B cells (red) compared with CD21+ B cells (black) in a patient with cGVHD.

We identified an expanded population of CD19+CD21− B cells (median 12.2, 1.3–34.9%) in the PB of patients with chronic GvHD. In contrast, significantly fewer CD19+CD21− B cells were present in the PB of patients without cGvHD (2.12, 0.67–20.1%; *p* = 0.004) and HC (3, 1.5–8.15%; *p* = 0.01); Figure 2A. Notably, the only patient without cGvHD with increased frequencies of CD19+CD21− B cells had evidence of EBV reactivation at the time of analysis.

We sought to further investigate the nature of the expanded CD21− B cell population in cGvHD patients in our study. Although expansion of CD21− B cells in cGvHD has been previously reported, the authors concluded that these cells are immature/transitional B cells (16). We performed a comprehensive phenotypic characterization of CD19+CD21− B cells in our cGvHD patients based on the expression of CD24, CD38, IgM, IgD, CD20, CD10, and CD27. The majority of CD21− B cells in the PB of cGvHD patients expressed high levels of CD20, lacked expression of CD10, CD27, CD24, and CD38 and expressed lower IgD and IgM than CD21+ B cells, a profile similar to that of exhausted B cells described in patients with chronic HIV viremia (26), while only a minority displayed the phenotype of transitional B cells [defined as CD24hiCD38hiIgMhiIgD+CD10+CD27− (30)], Figures 2B–D.

### Phenotypic Characterization of CD19+CD21− B Cells in cGvHD Patients

**Figure 3:**
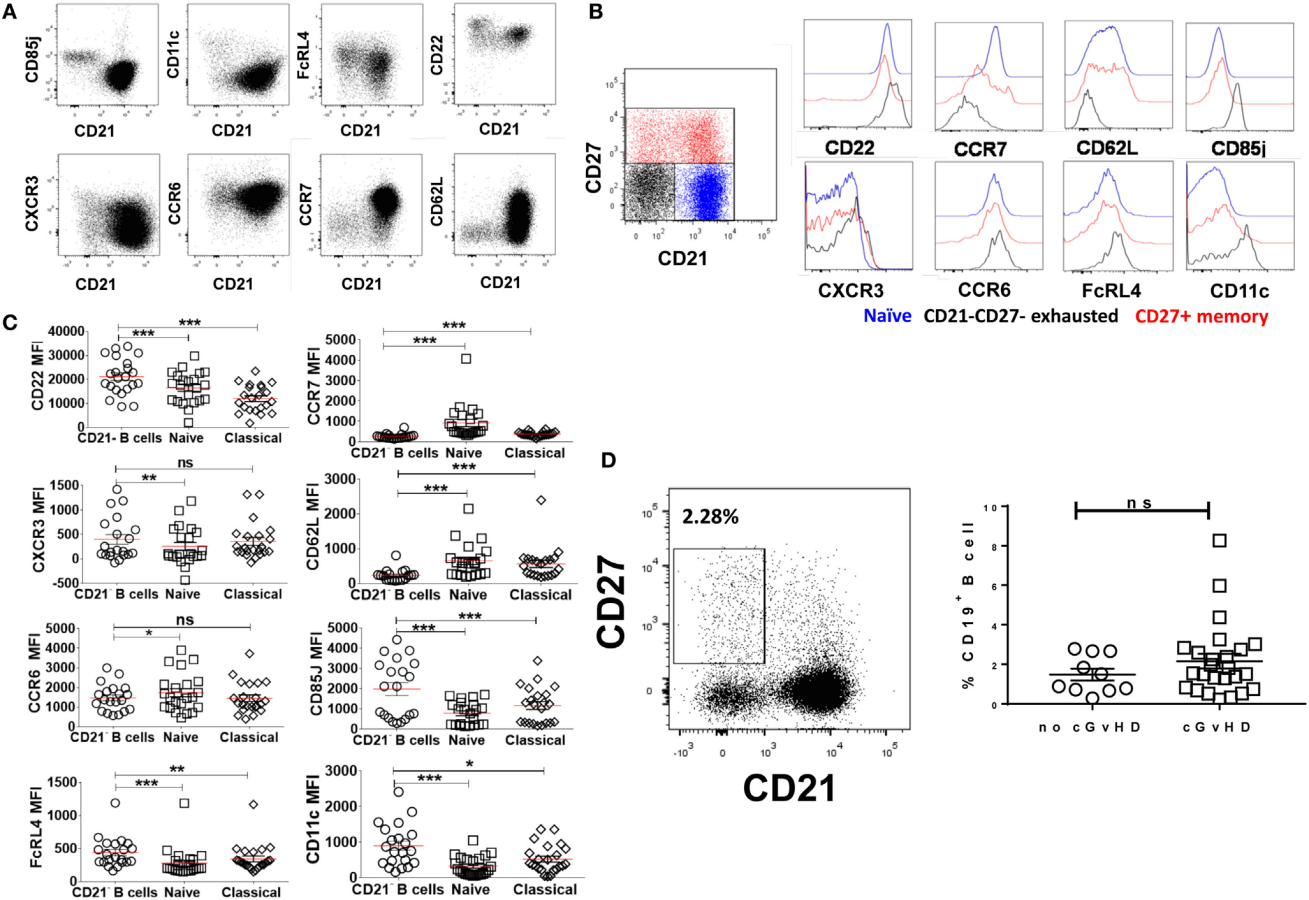
Expression of inhibitory and chemokine receptors on CD19+CD21−CD27−CD10− B cells. **(A)** FACS plots from a representative chronic graft-versus-host disease (cGvHD) patient show the expression of chemokine and inhibitory receptors on gated CD19+ B cells in relation to CD21. **(B)** Color-coded gating on CD27+CD21+ in B cells from a representative cGvHD patient. The expression of inhibitory and chemokine receptors on naïve (blue), classical memory (red), and CD21− B cells (black). **(C)** Inhibitory and homing receptor expression on CD21−CD27−CD10− B cells relative to naïve and classical CD27+ memory B cells. Plots depict individual median fluorescence intensity (MFI) values for each cell subpopulation from 22 cGvHD patients. **(D)** FACS plot show gating strategy on CD21−CD27+ activated memory B cells from a representative cGVHD patient (left). Scatter blot compare the frequency of activated memory B cells between the two post-transplant groups; *p* = ns. *Represents a *p* < 0.05, **p < 0.01, and ***p < 0.001.

The phenotypic similarities between the expanded CD21− B cell population in cGvHD and exhausted B cells in patients with chronic HIV viremia led us to seek a more precise phenotypic definition of CD20hiCD10−CD27−CD21− in cGvHD patients. Exhausted B cells in HIV are defined by the differential expression of an array of chemokine and inhibitory receptors (26), including upregulation of the inhibitory receptors Fc receptor-like protein 4 (FCRL4), CD22, and leukocyte immunoglobulin-like receptor, subfamily B (CD85J), and altered expression of the chemotaxis receptors integrin alpha X (CD11c), CXCR3, CCR6, selectin L (CD62L), and CCR7 (31, 32). Accordingly, we explored the expression of these markers on the expanded CD21− B cell population against naïve and classical memory B cells in 22 patients with active cGvHD and 10 no cGVHD patients; Figures 3A,B.

CD19+CD10−CD21−CD27− B cells in cGvHD, Figure S1 in Supplementary Material, expressed significantly higher levels of CD22, CD85J, and FCRL4 compared with naïve (CD19+CD10−CD21+CD27−) and classical memory B cells (CD19+CD10−CD21+CD27+); Figure 3C. Furthermore, CD21− B cells in these patients displayed a profile of altered trafficking and homing receptors, including significantly higher expression of CD11c and CXCR3 compared to naïve and classical memory B cells (*p* = 0.008 and *p* = 0.03 and *p* = 0.007 and *p* = 0.42, respectively). In contrast, the homing receptors CCR7 and CD62L were expressed at significantly lower levels on CD21− B cells compared with both classical memory and naive B cells (*p* = 0.007 and *p* = 0.008, respectively). Little difference was observed in the expression of CCR6 among the three B cell subsets; Figure 3C. In contrast, CD21− B cells form HSCT patient with no cGVHD expressed lower levels of CD22, CD62L, and CD11c compared with both naïve and memory B cells (*p* = 0.01, 0.004, and 0.02, respectively). We did not observe a significant difference in the expression of CD85J or FCRL4 between different B cell subsets (data not shown). Of note, there was no significant difference in frequencies of CD21−CD27+ activated memory B cells between the two posttransplant groups, Figure 3D.

Overall, these data indicate that CD21−CD10−CD27− B cells in cGvHD have altered homing and an inhibitory profile similar to that previously described for exhausted B cells in chronic viral infections (26, 31, 32).

### B Cells from cGVHD Patients Exhibit Impaired Proliferative Potential to B Cell Receptor (BCR) and CD40-Mediated Activation

To further investigate the hypothesis that B cells in cGvHD are exhausted, we compared the proliferative potential of CD19+ B cells in response to BCR engagement and CD40 triggering in patients with or without GvHD and HC. CFSE-stained PBMCs from 10 cGvHD, 7 patients without cGvHD, and 10 HC were stimulated with anti-CD3/anti-CD28 beads [T cells upregulate CD154 receptor (CD40L) upon stimulation, which in turn activates CD19+ B cells through CD40 triggering], anti IgM/anti IgG (BCR triggering), or the combination of anti-CD3/anti-CD28 and anti IgM/anti IgG for 96 h (provides a dual mechanism of B cell stimulation) (33).

**Figure 4:**
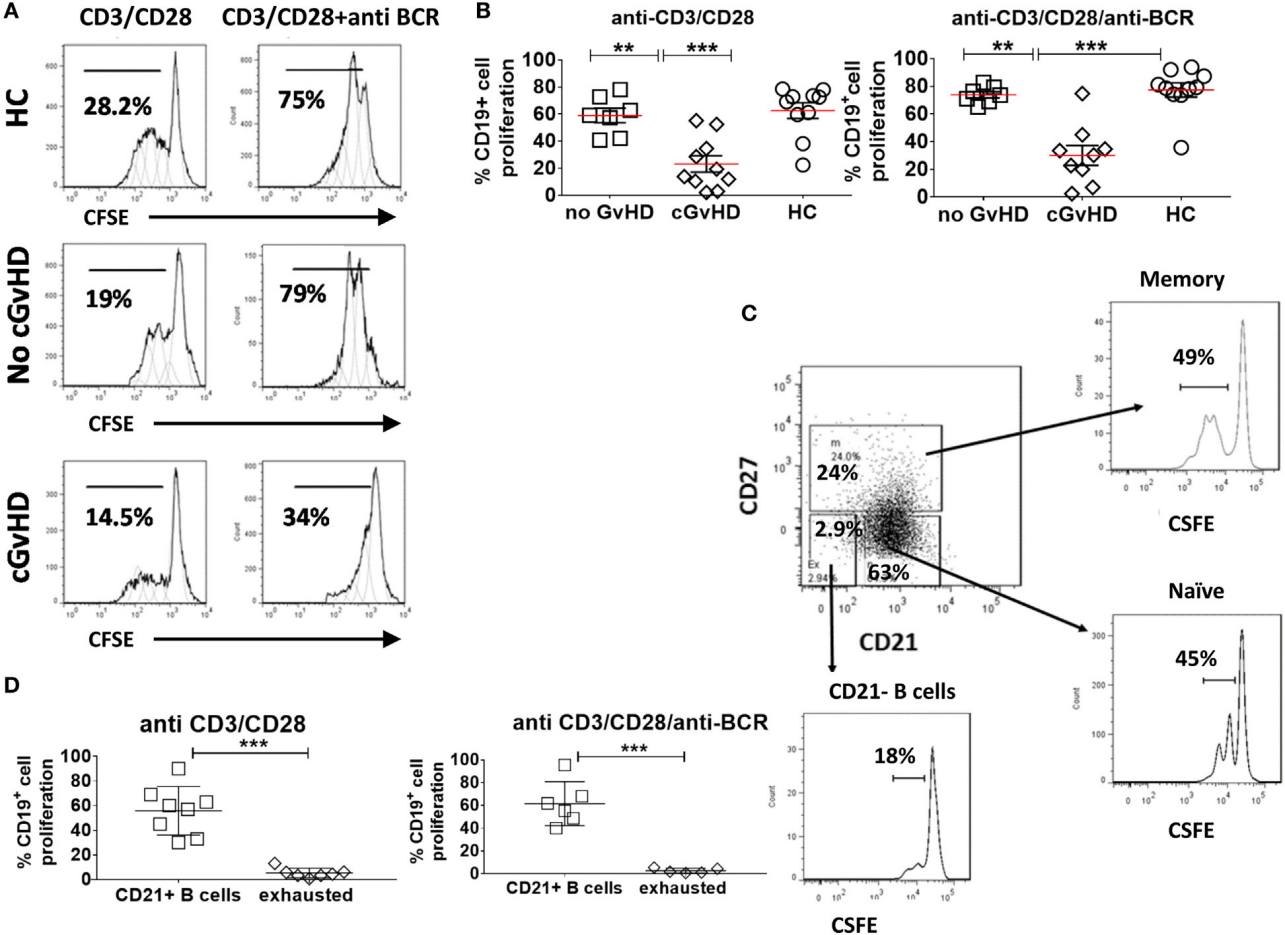
Proliferation of CD19+ B cell in response to B cell receptor (BCR) triggering and CD40L ligation. Carboxyfluorescein Succinimidyl Ester (CFSE)-stained peripheral blood mononuclear cells from healthy donors and patients with or without chronic graft-versus-host disease (cGvHD) were stimulated, anti-CD3/CD28 alone, or a combination of anti-BCR and anti-CD3/CD28 beads for 96 h. **(A)** Representative CFSE histograms comparing the proliferation of gated CD19+ B cells. **(B)** Comparison of B cell proliferation in 10 cGvHD patients, 7 no GvHD patients, and 10 healthy controls (HC). Chronic GvHD patients had the lowest proliferative potential in response to B cell stimulation compared with “no GvHD” patients and HC. **(C)** FACS plots of a representative cGvHD patient comparing the proliferation of CD27+ memory B cells and CD21+CD27− naïve B cells with CD21− B cells. **(D)** CD21− B cells proliferated significantly less than the rest of B cells (*n* = 8) when compared using non-parametric *t*-test *p* < 0.001.

The B cell proliferative response to stimulation *via* CD40 triggering alone (anti-CD3/CD28) or dual CD40 and BCR triggering was significantly lower in cGvHD patients compared to HC and patients without cGvHD patients [median percentage of dividing cells (16.5 versus 70.75 versus 59%; *p* = 0.0009) and (30.3 versus 79 versus 73.6%; *p* = 0.003), respectively], Figures 4A,B. We found no significant difference in the B cell proliferative response to dual CD40 and BCR triggering in patients with no cGVHD and HC (*p* = 0.14 and *p* = 0.037). Analysis of gated B cell subsets, from 10 patients with cGVHD revealed that the CD21− B cell subset proliferated less in response to stimulation with CD40 only or to dual CD40 and BCR triggering than the rest of CD21+ B cells (naïve and memory) (median 4.4 versus 58.5% *p* = 0.001), and (median 1.9% versus 58.6, *p* = 0.0003), respectively, Figures 4C,D, pointing to their inherently exhausted state.

These data indicate that the CD21−CD19+ B cell population in cGvHD exhibit proliferative deficiencies when compared with their CD21+ B cell counterpart and with B cells from patients without cGvHD or HC.

### Calcium Flux Is Impaired in Exhausted CD21− B Cells from cGvHD Patients

**Figure 5:**
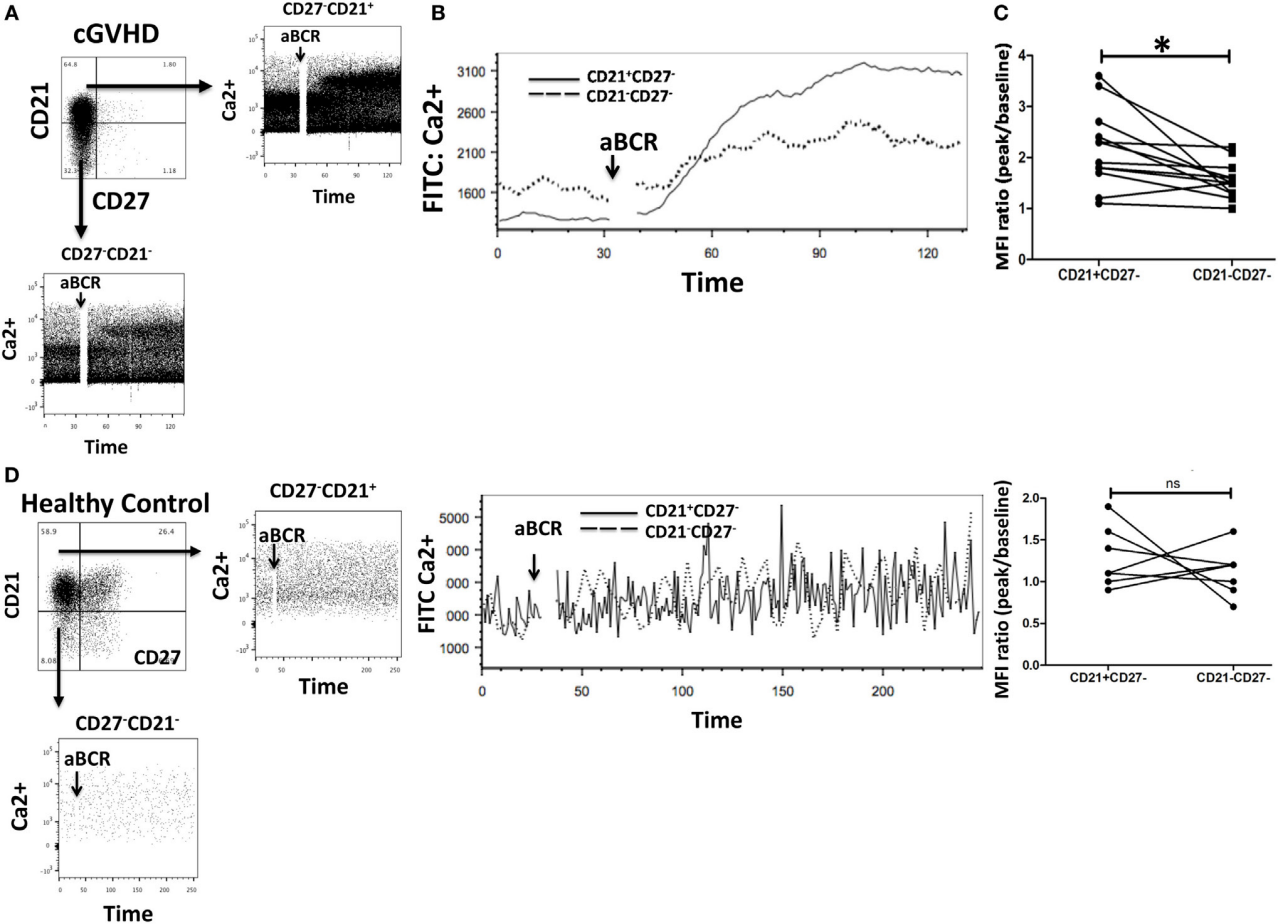
Exhausted B cells (CD21−CD27−) fail to mobilize calcium (Ca2+) in response to B cell receptor (BCR) triggering. **(A,B)** Peripheral blood mononuclear cells from chronic graft-versus-host disease (cGVHD) patients were loaded with the calcium dye Fluo-4AM and stained with monoclonal antibodies against CD19, CD21, and CD27 (*n* = 10). Ca2+ mobilization was assessed by flow cytometry after gating on CD21−CD27− and CD21+CD27− B cell subsets. CD21−CD27− B cells had an attenuated Ca2+ response compared to CD21+CD27− B cells. **(C)** Median fluorescence intensity (MFI) of Fluo-4AM was significantly lower in CD21−CD27− B cells (*n* = 10); *p* = 0.005. **(D)** CD21− B cells from healthy donors did not have reduced Ca2+ mobilization in response to BCR stimulation when compared with CD21+ B cells (*n* = 8; *p* = 0.15).

To investigate calcium signaling in B cell subsets in chronic GvHD, intracellular calcium levels were measured by flow cytometry in gated populations pre- and poststimulation of the IgM receptor in 10 patients with cGvHD and 8 HC. CD27−CD21− B cells from chronic GvHD patients had a reduced Ca2+ mobilization capacity compared to their CD21+ B cell counterpart (*p* = 0.005) Figures 5A–C. Interestingly, this was not the case when CD21−CD27− B cells (mainly transitional B cells) from HC were compared with CD21+ B cells (*p* = 0.147), indicating that reduced Ca2+ mobilization is specific to the exhausted CD21− B cell population (Figure 5D). Overall, these data suggest that CD21− B cells from cGvHD patients are anergic to BCR-mediated stimulation, in keeping with their exhausted phenotype and reduced proliferative potential.

### Expansion of CD21−CD27−CD10− B Cells Correlates with cGvHD

**Figure 6:**
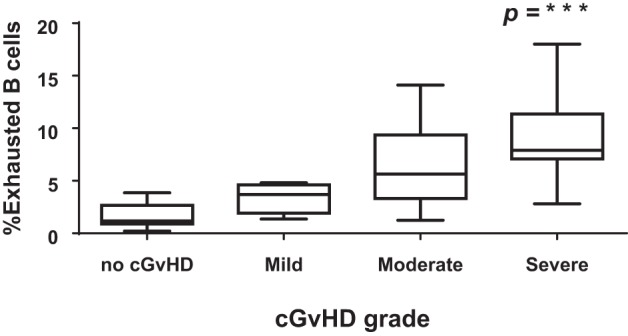
CD21−CD27−CD10− B cells and severity of chronic graft-versus-host disease (cGvHD). Box plot depict the severity of cGvHD (moderate to severe) according to CD21− B cell frequencies in 32 patients with cGVHD.

To determine if the exhausted (CD19+CD21−CD27−CD10−) B cell frequencies after HSCT were associated with cGvHD severity, we classified patients according to the frequency of CD19+CD21− CD27−CD10− B cells using a cut-off of 4% (upper limit of normal) (26). There was a significant association between the frequencies of exhausted B cells and the severity of cGvHD (Figure 6; *p* < 0.0001). We subsequently examined the association between cGvHD (none versus any) and other clinical variables (Table 1). Variables found to be significant at a *p* < 0.1 (CD19+CD21−CD27−CD10− frequency, underlying disease, stem cell source, and donor) were included in a logistic regression model. CD19+CD21−CD27−CD10− frequency >4% (OR = 158, 95% CI 10.2–2,441, *p* < 0.001), disease group (OR = 25.9, 95% CI 2–345, *p* = 0.04), and years from transplant (<2 versus >2 years) (OR = 10.6, 95% CI 1–108.9, *p* = 0.048) were found to be independently associated with cGvHD. Donor type was not significantly associated with cGVHD severity in any multivariate model. To account for the discrepancy in sample time posttransplant for patients with or without cGvHD, we carried out a logistic regression analysis but only for a sample days matched cohort (*N* = 38). As before, CD19+CD21−CD27−CD10− frequency >4% (OR = 47.7, 95% CI 4–518, *p* = 0.001) and disease group (OR = 18, 95% CI 1.4–226, *p* = 0.025) were found to be independently associated with cGvHD.

## Discussion

In this study, we further characterize the previously described expanded population of CD19+CD21− B cells in the PB of patients with active cGvHD and show for the first time that these cells bear the phenotypic signature of exhaustion (26), including increased expression of multiple inhibitory receptors, altered expression of homing receptors, a reduced proliferative potential, and a significant defect in calcium influx. The frequency of these cells was independently associated with the severity of cGvHD, providing evidence for cGvHD-associated B cell exhaustion.

Chronic GvHD is associated with perturbed B-cell homeostasis (22, 34, 35). Recent studies have reported CD19+CD21− B cells as a predictive biomarker of cGVHD (25) and associated with a higher risk of BOS (24). The authors identified these cells as transitional B cells (16, 23). Interestingly, in some patients with BOS, CD21− B cells were CD38loCD10− and lacked CD27 co-expression. Expanded CD19+CD21− B cells have also been observed in a number of conditions associated with chronic inflammation, including autoimmune disorders (36, 37), chronic infection (26, 29, 32, 38), and combined variable immune deficiency (39). Recent studies report the induction of a distinct subset of Tbet+ B cells in HIV infection that are maintained by chronic HIV viremia. Tbethi B cells were characterized by expression of immunoglobulin within both activated CD21−CD27+ memory cells and CD21−CD27− tissue-like memory B cells (40, 41). Indeed, the only patient in our study without cGvHD who had increased frequencies of CD19+CD21− B cells had evidence of EBV reactivation at the time of PB collection. However, to date, limited phenotypic and functional characterization of CD19+CD21− B cells has been performed in cGvHD. Low or absent CD21 expression is a characteristic of a number of B cell subsets including, transitional B cells (CD10+CD27−), plasmablasts (CD10−CD27hiCD20−), activated memory B cells (CD10−CD27+CD20+), and the recently characterized tissue-like memory exhausted B cells (CD10−CD21−CD27−) (26). Transitional B cells are defined by high expression of CD24 and CD38, expression of CD10 and absence of CD27 (30, 42, 43). Based on this definition, we show that CD19−CD21− B cells in our cGvHD patients are not transitional B cells and, instead, display a phenotype reminiscent of tissue-like exhausted memory B cells. These data support previous reports that transitional B cells are not increased in cGvHD (8, 19).

The CD19/CD21 complex is an essential B-cell coreceptor that functions synergistically to enhance signaling through the BCR in response to T cell-dependent stimulation (44, 45). In addition to its important role in the regulation of mature B cell activation, CD21 is also involved in germinal center formation and regulation of programmed death of anergic cells with low antigenic affinities (44, 45). Therefore, downregulation of CD21 on PB B cells is likely to result in impairment in normal B-cell functions such as activation of naïve B cells, germinal B cell survival, and persistence of B-cell memory. In addition, downregulation of CD21 is likely to raise the activation threshold of B cells and contribute to anergy.

To further characterize the PB-derived CD19+CD21− B cells in cGvHD, we used multi-parameter flow cytometry to assess the expression of a panel of surface markers. The majority of CD19+CD21− B cells in the PB of cGvHD patients expressed high levels of CD20 and low levels of CD27 and CD10. Furthermore, the expanded CD19+CD21−CD20hiCD27−CD10− B cell population in cGvHD patients expressed relatively high levels of the inhibitory receptors CD22, CD85J, and FCRL4. These cells exhibited an altered profile of chemotaxis receptors, including higher expression of CD11c and CXCR3, which may contribute to their preferential localization to inflammatory sites, and lower levels of CCR7 and CD62L (l-selectin), homing receptors that facilitate B cell migration to lymph nodes (46, 47). Such alterations are similar to the signature of exhausted B cells described in chronic HIV viremia (26). Collectively, these data suggest that CD19+CD21− B cells in cGvHD are likely to preferentially migrate from lymphoid tissues to cGvHD inflammatory sites.

To investigate the hypothesis that CD21− B cells in cGvHD patients are exhausted, we evaluated their proliferative potential and their capacity to mobilize calcium in response to activation. Compared to B cells from patients without cGvHD and HC, B cells from cGvHD patients, and especially the CD21− subset, proliferated significantly less in response to T-cell help in the form of CD40L alone or with BCR cross-linking. In keeping with these data, the CD21− B cell subset in cGvHD patients had an impaired calcium response to BCR stimulation compared to CD21+ B cells. In contrast, CD21− B cells from HC and patients without cGvHD patients proliferated efficiently, with no difference in their ability to mobilize calcium when compared with CD21+ B cells. These findings could be explained by the overexpression of inhibitory receptors by this subset of cells. Higher expression of the inhibitory receptors CD22, CD85J, and FCRL4 and lower expression of CD21 increase the activation threshold of B cells and reduce BCR-mediated signaling (45, 48–50). Conversely, silencing of FCRL4 in CD19+CD21− B cells restore their response to BCR-ligation (51). Taken together, the increased expression of multiple inhibitory receptors, altered expression of homing receptors, and their reduced proliferative potential suggest that this subset of B cells in cGvHD display an “exhausted” phenotype.

Chronic GvHD mimics autoimmune and fibrogenic conditions in which B cells are constantly exposed to self antigens as well as pathogen-associated molecules and, therefore, are in a persistent state of activation (20). These inflammatory environments activate B cells in both T cell dependent and independent manners, which could in turn lead to B cell exhaustion. A number of investigators have reported the presence of expanded populations of antigen-specific, clonal CD19+CD21−/lo B cells in the PB of patients with chronic viremia. These CD19+CD21lo B cells were anergic, with decreased proliferation and attenuated calcium response to BCR stimulation (26, 29, 52). It is, therefore, conceivable that B cell exhaustion in cGvHD is driven by chronic antigenic stimulation and that, in order to prevent self-destruction and autoimmunity, activated B cells employ mechanisms such as downregulation of CD21 and upregulation of inhibitory receptors to dampen BCR-mediated activation. There is some evidence that B cell exhaustion in chronic HIV viremia might be antigen driven (26). In addition, chronic non-specific T-independent activation of B cells induced by HIV-associated molecules such as gp-120 in the presence of high BAFF levels has also been shown to cause functional exhaustion (53).

It is not clear whether exhausted CD19+CD21− B cells in cGvHD are directly involved in the disease pathogenesis, or are a consequence of continuous activation in the inflammatory cGvHD environment or alternatively, develop as a result of impaired B cell reconstitution. Without better tools to analyze antigen-specific B cells at the single-cell level, and a better understanding of antigens involved in the B cell-driven immune process in cGvHD, many of the concepts regarding the mechanisms of B cell exhaustion in cGvHD remain speculative.

A recent study reported that downregulating the expression of inhibitory receptors on exhausted CD21− B cells using RNAi technology can effectively reverse B cell exhaustion in HIV and improve immunity against pathogenic organisms (51). It is tempting to suggest that downregulating inhibitory receptors on CD19+CD21− exhausted B cells in cGvHD may also improve their effector function and at least partially reverse the state of immune impairment seen in these patients. Alternatively, if CD21− B cells are in fact antigen-specific and directly involved in the pathogenesis of GvHD, reversing their state of anergy may have deleterious consequences on disease severity. The observation by our group and others of higher frequencies of CD19+CD21− B cells in the PB of patients with more severe active cGvHD (16) supports a causal relationship between this unusually expanded subset of B cells and cGvHD. Furthermore, it is possible that chronic antigenic stimulation or T cell-independent B cell activation in the presence of elevated BAFF levels may contribute to B cell exhaustion (53).

To summarize, we report the presence of an expanded population of exhausted CD19+CD21−CD27−CD10− B cells in patients with active cGvHD. Evidence for B cell exhaustion included increased expression of multiple inhibitory receptors, altered expression of homing receptors, and a reduced proliferative potential. These data provide a possible explanation for the inadequacy of the antibody response against pathogens and the state of immunodeficiency observed in cGvHD (54). We conclude that CD19+CD21−CD27−CD10− B cells may serve as a potential biomarker to assess the severity of cGvHD and may provide a potential target for therapy.

## Ethics Statement

All patient samples were collected after written informed consent, according to local policy guidelines and in accordance with the declaration of Helsinki.

## Author Contributions

AK performed experiments, participated in the design and interpretation of the analysis, and wrote the manuscript. AA, RF, CS, KK, and EG performed experiments, participated in interpretation of the analysis, and commented on the manuscript. AMA, MM, HS, NC, RM, JFA, RC, and ES provided advice on experiments and data analysis and commented on the manuscript. DM and RS commented on the manuscript and performed statistical analysis. KR designed and directed the study and wrote the manuscript.

## Conflict of Interest Statement

The authors declare that the research was conducted in the absence of any commercial or financial relationships that could be construed as a potential conflict of interest.
