# Supplementary material for: Evidence for B Cell Exhaustion in Chronic Graft-versus-Host Disease
Source: Front Immunol. 2018 Jan 12;8:1937. doi: 10.3389/fimmu.2017.01937 (PMC5770573; doi:10.3389/fimmu.2017.01937)
Supplement: Supplementary file 1 [file Presentation_1.pptx]

## Slide 1
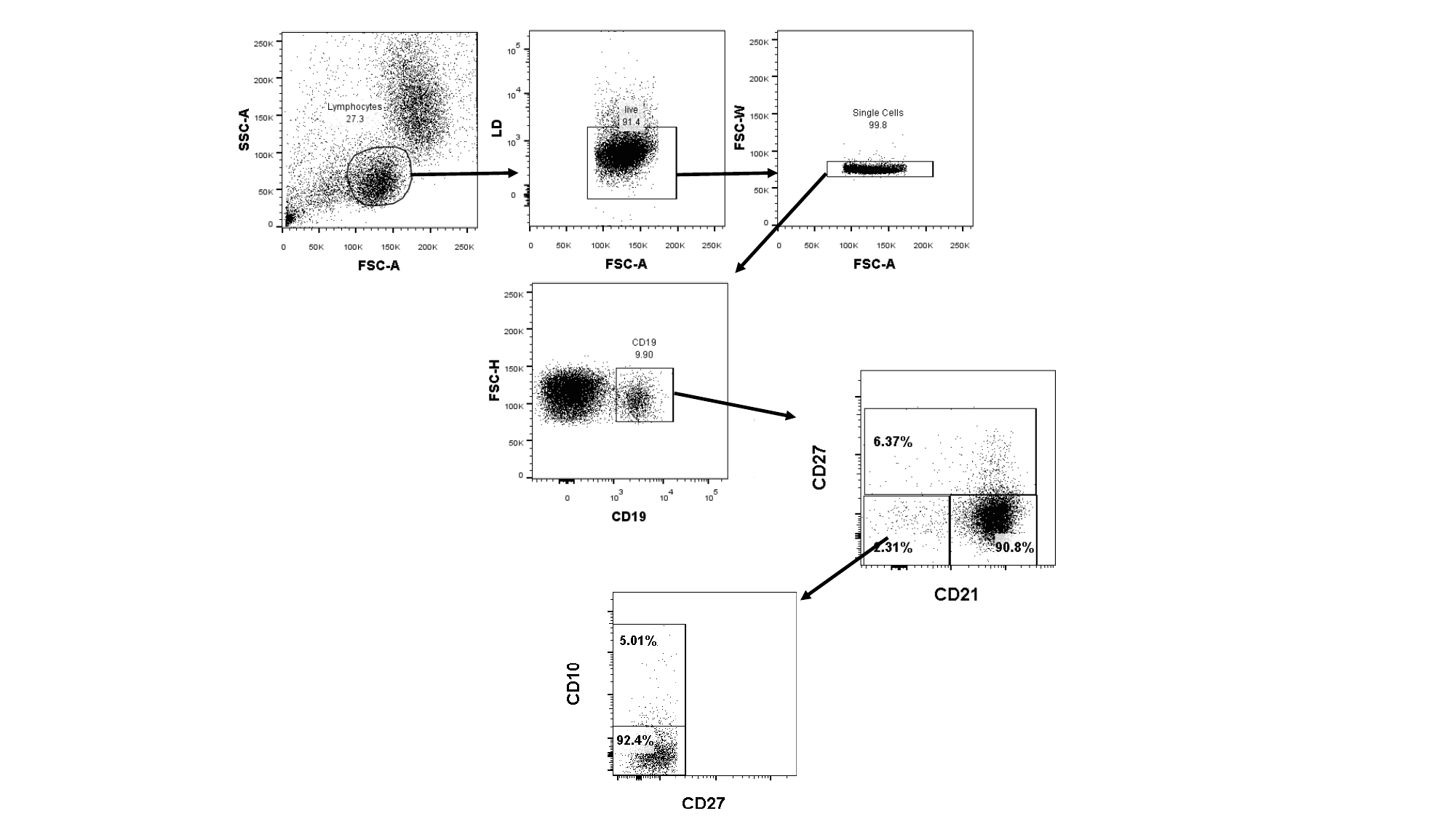

## Slide 2
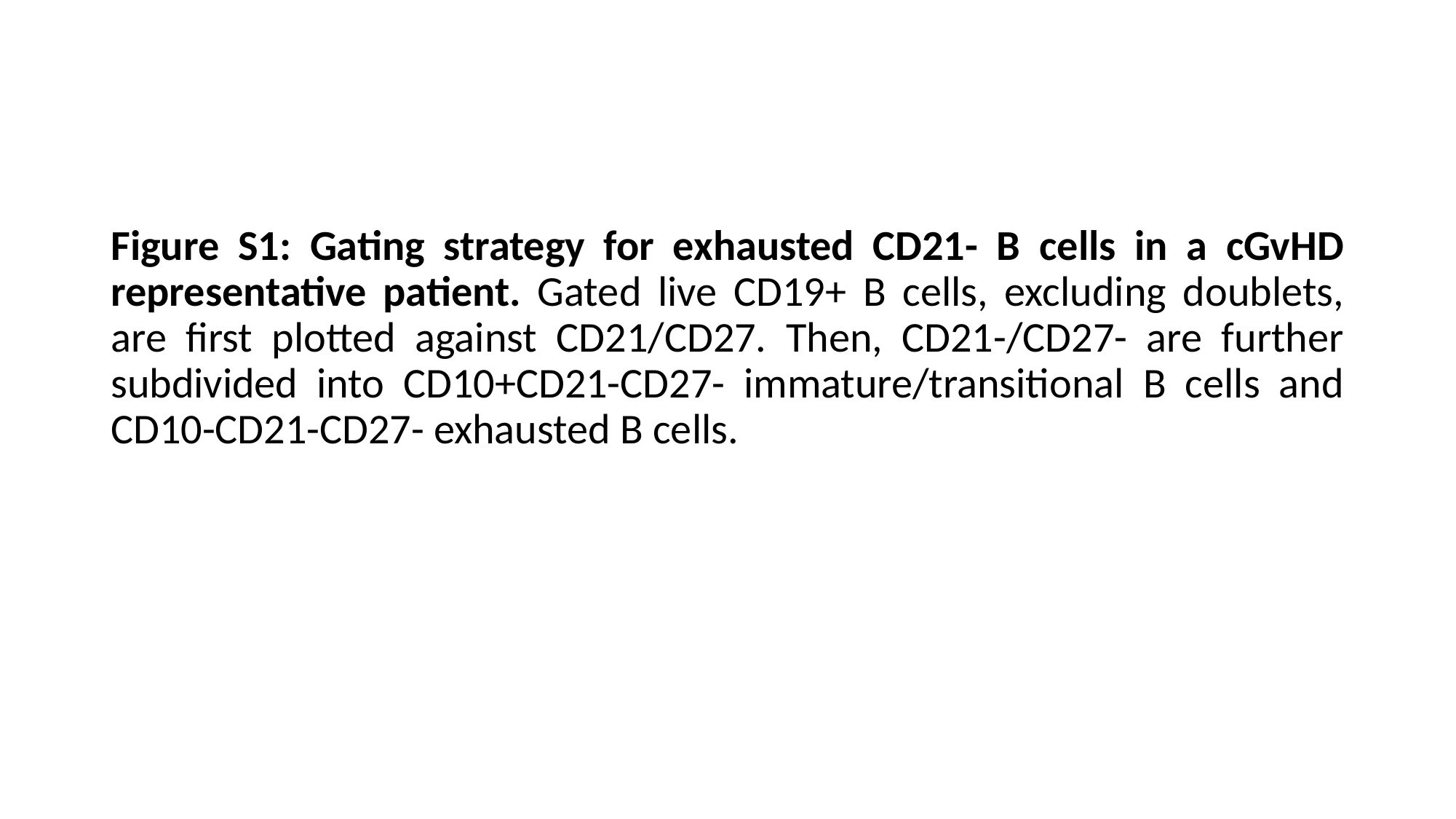

Figure S1: Gating strategy for exhausted CD21- B cells in a cGvHD representative patient. Gated live CD19+ B cells, excluding doublets, are first plotted against CD21/CD27. Then, CD21-/CD27- are further subdivided into CD10+CD21-CD27- immature/transitional B cells and CD10-CD21-CD27- exhausted B cells.
